# A Content Analysis of Online Messages about Sugar-Sweetened Beverages

**DOI:** 10.3390/nu16071005

**Published:** 2024-03-29

**Authors:** Yingke Li, Lijiang Shen, James Price Dillard, Shu (Scott) Li

**Affiliations:** 1 Department of Communication Arts & Sciences, The Pennsylvania State University, State College, PA 16802, USAjpd16@psu.edu (J.P.D.); 2 School of Communication, The University of Akron, Akron, OH 44325, USA

**Keywords:** sugar-sweetened beverages, sugary drinks, content analysis, video messages, perceived effectiveness

## Abstract

Media campaigns can reduce or promote the consumption of sugar-sweetened beverages (SSBs). Brief, US-based English-language online messages were gathered from searchable media platforms, a process that yielded 112 anti-SSB videos and 29 pro-SSB commercials. Using a combination of inductive and deductive methods, a content analysis of those messages was conducted to identify their properties. They were coded for the direction (pro vs. anti), target of the advocacy (e.g., consumption vs. policy), actor demographics (gender, age, and ethnicity), persuasive theme (e.g., excessive sugar, nurturing), and message sensation value. Anti-SSB appeals primarily targeted individual-level consumption behavior. They utilized six persuasive themes and often included more than one theme in a single message. Pro-SSB messages used feel-good themes and utilized only one theme per message. The proportions of adults, adolescents, and children differed by the direction of the advocacy. Black, Hispanic, and Asian actors were under-represented in the anti-SSB sample relative to Whites. Pro-SSB appeals were slightly higher than anti-SSB appeals in message sensation value (*p* = 0.09). The findings illuminate the message features that characterize the universe of brief anti-SSB appeals available online, highlight messaging disparities, and reveal the absence of certain common, effective persuasive themes.

## 1. Introduction

Sugar-sweetened beverages (SSBs) are drinks that include added sugars: non-diet soda, fruit drinks, sports and energy drinks, and sweetened coffee and tea [1]. Such drinks are consumed quite frequently. Roughly two-thirds of Americans consume one or more SSBs every day [2,3,4]. Although SSB consumption has declined somewhat over the last two decades, it remains quite high [3]. These levels of consumption are not without consequences. SSBs are causally implicated in a variety of negative health conditions including weight gain, obesity, and type 2 diabetes [5,6] as well as various forms of cancer [7,8,9,10].

To counter the effects of commercial advertising, health advocates have turned to mass media campaigns. Often, these efforts have proven to be successful at persuading members of the public to reduce consumption [11]. Yet, because each campaign is conducted independently, the impact is also assessed individually, one campaign at a time [12,13]. Thus, little is known about these efforts collectively. This is unfortunate in that systematic knowledge of anti-SSB messages as a group would improve understanding of consumption reduction efforts more broadly. What is needed is a description of the properties of SSB campaign messages and the frequency of occurrence of those properties.

Content analysis is the appropriate tool for this task. It is a descriptive research technique designed to isolate concepts and themes that are present in qualitative data. Once themes have been identified, the frequency of each is computed. The resulting forest-level (vs. tree-level) view can enable comparisons with campaigns in other health domains (e.g., smoking) [14]. In addition, it lays the groundwork for the subsequent analysis of message effectiveness as a function of message features, such as persuasive strategy.

Given the lack of a broad, systematic understanding of efforts to reduce SSB consumption via media campaigns, the objective of this research was to conduct a content analysis of anti-SSB public service announcements. To provide a point of comparison, we also collected and analyzed a sample of commercial pro-SSB advertisements. The analysis focused on features of persuasive messages including the target of change (e.g., consumption or policy), demographic features of the actors (e.g., gender), persuasive strategy (e.g., health consequences), and on one stylistic index known as message sensation value.

This report is structured as follows: (a) a review of the literature aimed at summarizing prior research on the features of SSB messages, (b) a detailed description of the methods used to carry out the content analysis, (c) the presentation of the results, and (d) a consideration of the implications of the findings for public health efforts to reduce SSB consumption.

### Message Features

Content analysis intermingles top-down and bottom-up thinking. The first type of logic calls for establishing facts, to the extent that they are known. Accordingly, the first step was a preliminary review of codable message features. Based on theory, research, and our personal knowledge of the context, we considered four categories of message variables. The first category considered features inherent to all persuasive appeals: the direction and target of the advocacy. With respect to SSBs, the directional aspect of the advocacy is obvious: public health advocates design messages to steer individuals away from sugary drinks. These are anti-SSB messages. In contrast, the beverage industry devotes enormous sums of money to the production of messages designed to create favorable impressions of their products [15]. These are pro-SSB messages. Although our primary interest was anti-SSB messages, a comparison with pro-SSB messages could provide insight into the competitive SSB message environment.

The target of a persuasive message is the attitude, action, or policy that the message advocates for or against. One obvious target is consumption behavior, but others were likely to be present in the SSB environment too. For instance, public health efforts sometimes take aim at policy-level changes such as the implementation of SSB taxes, limiting portion sizes, or creating age-based restrictions on SSB sales [16]. We sought to develop a compact but comprehensive set of codes for these fundamental features of persuasive messages. Given the absence of theory and the inductive aspects of content analysis, hypotheses (i.e., predictions derived from theory) would be both inappropriate and impossible. As is consistent with the method, we posed research questions.

Research Question 1: What set of fundamental message features (i.e., the direction and target of the advocacy) characterize anti- and pro-SSB messages and how frequently does each occur?

The gender, age, and ethnicity of audience members are commonly assumed to be key segmentation variables. Yet the impact of persuasive messages is determined not by these variables alone but by the similarity between audience members and actors. There is evidence that audience–actor matching enhances the effectiveness of messages advocating for HIV prevention behaviors [17], for smoking reduction [18], against overeating [19], and for alcohol consumption reduction [20]. Given the widespread knowledge of this effect, it seemed reasonable to suppose that actor demographics provide insight into the intended audiences of anti- and pro-SSB messages.

Research Question 2: How frequently do actors of varying gender, age, and ethnicity appear in anti- and pro-SSB messages?

Persuasive messages can be characterized in terms of strategy [21]. For example, Brownbill and colleagues’ [22] analysis of pro-SSB marketing to Australians on Facebook revealed that most manufacturers’ messages exhibited themes of sporting prowess, masculinity, and engagement with the outdoors. The one exception was Coca-Cola, whose advertisements emphasized “fun, happiness, and friendship” (p. 357). Research on the other side of the pro–con divide can be found in Bleakley et al.’s [23] assessment of 26 anti-SSB messages. Those investigators identified fear, humor, and nurturance/caretaking as distinct themes. While maintaining attention to prior work, we also remained open to the possibility that additional themes might be found in larger samples of messages. We asked the following:

Research Question 3: What persuasive themes appear in anti- and pro-SSB messages and how frequent is each?

Messages also vary in terms of style. Message sensation value (hereafter MSV) is a stylistic message feature based on objective message components such as visceral images, sound saturation, and special effects [24]. It may be understood as a summary variable that captures an intensity type of effect that is the product of variations in style. We viewed it as potentially informative for a few reasons. One, there is evidence that it plays an important role in different portions of the persuasive process. It attracts attention and amplifies emotional responses [25] while also impeding learning and retention [26]. Another reason for our interest is that previous research has assessed MSV in other health domains [27,28], offering points of comparison with our SSB data.

Research Question 4: What levels of MSV are present in anti- and pro-SSB messages and how do they compare to other health domains?

## 2. Materials and Methods

Content analysis is a systematic method for quantifying qualitative data. In this project, the qualitative data of interest were video messages on the topic of SSBs. Thus, the first task was to gather a set of SSB-related messages. The second task was to perform the translation from qualitative data to quantitative representations of those data. Achieving the latter goal required that we develop instrumentation (i.e., a coding scheme), train human coders in its application, and then evaluate their reliability. We address each of these issues below.

### 2.1. Message Collection

We collected anti- and pro-SSB messages using procedures that were unique to each message type. We describe those procedures next.

#### 2.1.1. Anti-SSB Messages

Our goal was to collect all English-language anti-SSB public service announcements (PSAs) that were (a) available online, (b) two minutes or less in length, and (c) produced in the U.S. To be clear, we did not seek to sample from the universe of messages within these parameters. Rather, we aspired to capture the entire universe.

During the spring of 2019, searches by our research team were conducted via Google, Bing, Facebook, Twitter, Instagram, and YouTube. At the time, TikTok did not have the major presence in the U.S. that it has today. Thus, it was plausible that search engines and social media platforms that we used were capable of identifying the universe of messages that we sought. The search terms were as follows: sugary drinks/beverages, sugar-sweetened drinks/beverages, anti-SSB, anti-soda, and anti-sugar, all of which were used alone and in combination with PSA and tax (plus soda tax). The search criteria were adapted to each platform due to variations in how messages were collected or organized. Platforms such as YouTube identified related messages (in the Up Next column), which enabled something akin to snowball sampling.

After eliminating duplicates, these procedures yielded 107 messages that advocated for the reduced consumption of SBBs and five messages that advocated for the implementation of a tax on SSBs (i.e., pro-tax messages). Although our goal was to gather all of the messages that met our criteria, it was not possible to independently verify whether or not we were successful. Without the firm knowledge of the boundaries of the population, we can only claim to have a (non-probability) sample of anti-SSB messages.

#### 2.1.2. Pro-SSB Messages

The population of pro-SSB messages is vast. For example, a Google search on soda advertisements (on 3 March 2024) yielded 310,000,000 results. Our goal was to develop a small but prototypical sample of pro-SSB messages for use in the message effects portion of this project. This necessitated a multi-pronged strategy that differed from our anti-SSB efforts. Accordingly, we gathered messages via three procedures. First, the research team used the same search engines as before to identify popular beverages in five categories: sweetened juice, soda (not diet), sweetened tea/coffee, sports drinks, and energy drinks. Searches were conducted by coupling each of these terms with advertisement. Next, from the results, we chose high-ranking advertisements (i.e., those from the top of the search results) that varied in the manufacturer and product for a total of 22 messages. Given the relative prevalence of soda commercials, we selected six advertisements for soda and four for each of the other categories.

Our second procedure utilized the website of the American Beverage Association. The site contained seven pro-industry messages: (a) drink manufacturers care about the health implications of overconsumption, (b) consumers should have freedom of choice, and (c) drink taxes are undesirable. We retained all seven.

Finally, we followed links identified in the anti-SSB search that connected to anti-tax messages. When it became clear that only one anti-tax message could be obtained, it was eliminated from the database on the grounds that we could not draw category-level inferences from a single case. The combination of commercial advertisements and pro-industry messages gave a total of 29 pro-SSB messages. Although our procedures resembled stratified sampling, because of the use of non-overlapping subdivisions, readers should be aware that the sampling elements (i.e., messages) were not selected randomly within subdivisions. The procedures were not probability-based but rather more akin to a convenience sample. Nonetheless, given our procedures and inspection of the messages, we judged the set as a coherent and meaningful representation of the universe of pro-SSB messages at the time of sampling.

### 2.2. Coding Procedures

Our approach to coding combined deductive and inductive methods, the mix of which depended on the degree to which a plausible set of categories could be derived from pre-existing theory or research [29]. In the case of established concepts, such as the direction of the advocacy and MSV, this was largely a matter of applying that which was already well specified. Hence, no revision of codes was necessary. Coding in more novel domains, such as persuasive themes, involved iterative interaction with the data. This was a more emergent process.

**Figure 1:**
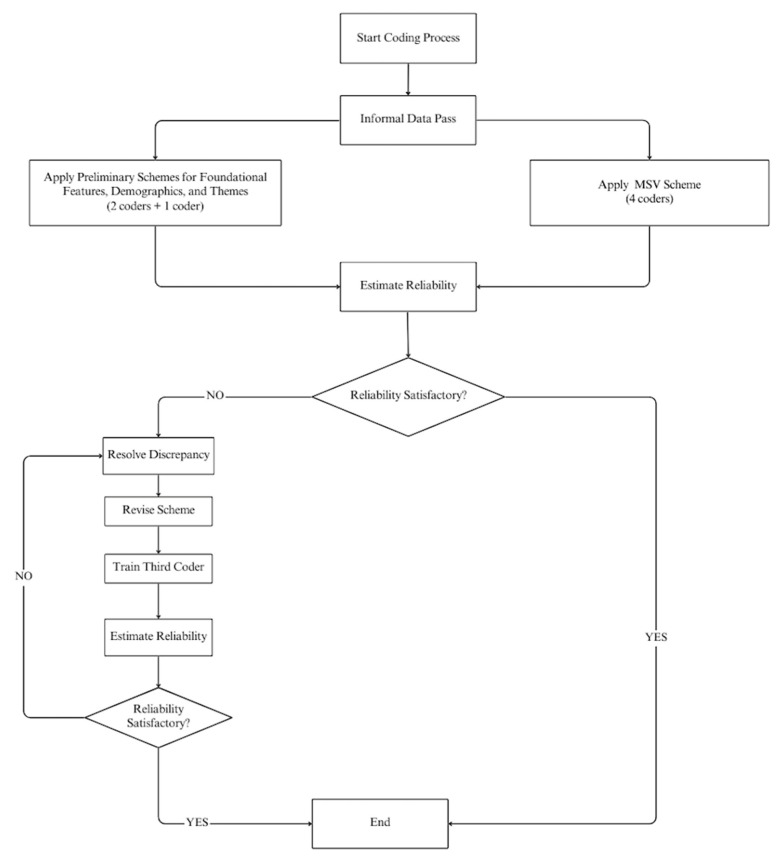
A flowchart of the coding process.

Figure 1 and the text in the next section explain these processes in a general manner. Following that, we provide the details for each of the four categories of codes.

To begin, we conducted an informal pass through the data to derive initial impressions of the viability of the coding categories (see Figure 1). Next, two coders applied the preliminary coding scheme to the data and estimated reliability using the categorical form of Krippendorf’s alpha [30,31] (which we represent as *αk*). If this produced satisfactory results, the coding process was considered complete.

When reliabilities proved inadequate, the two coders reviewed their discrepancies, revised the scheme, and then jointly applied it to the data, thereby producing a single set of classifications [32]. Then, a third coder was trained on the revised scheme using a subset of the messages. Reliability was estimated by comparing the third coder’s classifications to the joint classifications produced by the previous pair of coders. If reliability was satisfactory, differences were resolved by discussion and the coding process was considered complete. Given the large number of codes for message sensation value, we utilized four coders, each of whom classified three-quarters of the messages. Reliability was estimated for each pair of coders, then the average was computed.

### 2.3. Codes

#### 2.3.1. Fundamental Message Features

With respect to target, an initial review of the messages suggested that they emphasized consumption, regulation (e.g., taxation), or the SSB industry. And of course, messages might be either pro- or anti-SSB. To capture these variations, we initially created three mutually exclusive and collectively exhaustive codes. With respect to consumption, messages were classified as anti (i.e., opposed to consumption), pro (i.e., in favor of consumption), or neither. Regulation messages were anti-SSB (i.e., in favor of regulating SSBs), pro-SSB (i.e., against regulation), or neither. The industry categories included anti-SSB (e.g., “SSB companies have lied to you.”), pro-SSB (e.g., “We [the beverage companies] are working to give consumers the reduced sugar beverages that they want.”), or neither. Our first attempt at applying this scheme revealed that consumption and industry themes were often conflated in both pro- and anti-messages. Accordingly, we removed the exclusivity constraint and coded the data again. A summary of the categories, their associated codes, and reliabilities are given in Table 1. (The direction of the advocacy does not appear because it was part of our sampling procedure).

#### 2.3.2. Actor Demographics

In our informal first pass through the messages, two features of the data became apparent: (a) some messages had no actors at all and (b) efforts to code cartoon characters with respect to actor demographics were unlikely to be successful. When both sorts of messages were filtered out, k was reduced to 77. Next, we applied the coding scheme. Making binary judgments, two coders assessed the presence versus absence of each demographic feature. A summary of the categories, their associated codes, and reliabilities are given in Table 2.

#### 2.3.3. Persuasive Themes

Pro- and anti-SSB messages presented a variety of reasons to adopt their respective advocacies. Different sets of codes emerged for two message types, although both sets consisted of non-exclusive, present/absent codes.

For anti-SSB messages, six persuasive strategies were identified. The theme labeled health consequences reflected content that described one or more of the adverse medical conditions that were the product of SSB consumption (e.g., weight gain or obesity, cancer, Type II diabetes, and heart disease).

Messages that expressed an excessive sugar theme focused on the large quantity of sugar in SSBs (*αk* = 0.94). This was illustrated by, for example, children making sports drinks using different types of sugar and young adults concocting SSBs on the street using the same quantity of sugar that is present in industry-manufactured beverages.

The nurturing theme emphasized the importance of care-giving. Such messages mentioned parents caring for their offspring, making decisions about children’s SSB consumption, and taking action to protect their children.

We coded as humor messages those that made an effort to amuse viewers by using non-human characters or animations, making a joke, children acting like adults, or actions that were improbable (e.g., being hit with a giant glob of fat).

Industry manipulation messages centered on misleading information provided by members of the beverage industry and their irresponsible behaviors, which included their failure to take actions to address the health concerns of SSB overconsumption.

Expertise messages contained scientifically derived recommendations such as the recommended amount of added sugar per day. This category also included statements by healthcare professionals, educators, or policy makers that called for reducing SSB consumption.

For pro-SSB messages, we developed three non-exclusive strategies. Feel-good/celebrity utilized famous persons who expressed positive feelings for SSB products (*αk* = 0.80). Messages that focused on presenting positive feelings (e.g., happiness) when regular (non-celebrity) people drink sugary beverages were coded as feel-good/regular person. The industry promotion theme reflected claims regarding the cooperation of members of the SSB industry (and, e.g., the industry responsiveness to consumers’ desire for lower-sugar drinks). A summary of the categories, their associated codes, and reliabilities are given in Table 3.

#### 2.3.4. Message Sensation Value

Given the existing body of research on MSV, we expected that the existing coding scheme [24] would not require modification. This proved to be true. The average reliability estimates across pairs of coders for the nine MSV judgments are given in the right-most column of Table 4 along with the MSV element categories and their codes. So that each element would be weighted equally, all judgments were placed on a 0–1 scale, then a single score for message sensation value was created by averaging the nine codes. Thus, the final MSV index was on a 0–1 metric.

## 3. Results

### 3.1. Fundamental Message Features

Table 5 organizes the messages in terms of the direction and target of the advocacy. A lower-case k is used to indicate the number of messages. As shown, there were 112 anti-SSB messages, 94.6% of which were explicitly concerned with consumption. A smaller but nontrivial number (17.9%) took the SSB industry as their target. Only 3.6% were concerned with regulation, all of which focused on implementing an SSB tax.

The analysis utilized 29 pro-SSB messages, all of which endorsed consumption, either implicitly or explicitly. For the actor demographic and persuasive theme analyses, we reconfigured the data along two lines. First, because the fundamental features analysis identified only four regulation messages among the anti-SSB appeals, they were set aside on the grounds that generalizations based on four cases would be weak. Second, given that all the pro-industry messages also included a pro-consumption appeal, we collapsed across those two targets. These changes enabled apple-to-apple comparisons among the largest number of pro- and anti-SSB messages. For the actor demographic analyses only, messages with no characters or non-human characters were filtered out.

### 3.2. Actor Demographics

The results of the actor demographics analysis are given in Table 6. Focusing first on gender, anti-consumption messages were characterized by a nearly equal representation of males (84.89%) and females (81.4%). Conversely, a higher representation of males (96%) relative to females (68%) was observed among the pro-SSB messages. No statistically significant gender disparities were detected between pro- and anti- messages. Cross-tabs revealed that men and women were co-present in 52.7% of the anti-SSB messages and 55.2% of the pro-messages.

Turning to actor age, the data showed that roughly two-thirds of the anti-SSB messages included children and adults. Adolescents were present in slightly more than half of those messages. Relative to pro-SSB messages, both children and adolescents were over-represented in anti-SSB messages by differences of 32.0% and by 28.0%, respectively. Conversely, adults were over-represented in pro- versus anti-messages by 26.2%. Additional cross-tabs indicated that children and adolescents were co-present in 24.1% of anti-messages and 17.2% of pro-messages; children and adults appeared together in 42.9% of anti-messages and 24.1% of pro-messages; adolescents and adults were co-present in 28.6% (anti) and 24.1% (pro).

Regarding ethnicity, White and Black actors were present in the majority of both anti- and pro-messages. Hispanic actors appeared less frequently and Asian actors even less frequently. The only statistically significant cross-column comparison revealed that White actors were more common in pro-SSB versus anti-SSB messages, a difference of 21.2%.

Cross-tabs revealed that the most common co-presence occurred between White and Black actors across both advocacy directions: 36.6% of anti-messages and 48.3% of pro-messages. Co-occurrences of White and Hispanic actors and Black and Hispanic actors each accounted for 21.4% of anti-messages, followed by Black and Asian actors (18.8%), White and Asian actors (16.1%), and Hispanic and Asian actors (15.2%). In pro-messages, the co-presence of White and Hispanic actors was found in 27.6% of the sample, succeeded by Black and Hispanic actors (24.1%). The remaining three combinations—White/Asian, Black/Asian, Hispanic/Asian—were each present in 13.8% of the pro-messages.

### 3.3. Persuasive Themes

As noted above, there was no overlap in the persuasive themes present in the anti- versus pro-SSB messages. Consequently, Table 7 contains empty cells in both the anti- and pro-SSB columns. This means that the results can be understood to represent the degree to which one group differs from another as well as whether the frequency of any theme is significantly different from zero. All within-row comparisons indicated significant differences.

Of the six anti-SSB themes, excessive sugar was the most frequent, followed by nurturing, health consequences, expertise messages, industry manipulation, and humor. It was not uncommon to see messages composed of multiple themes. The most frequent co-occurrences were health consequences and nurturing (20.8%), followed by health consequences and expertise messages (16.0%), health consequences and excessive sugar (16.0%), excessive sugar and nurturing (12.3%), health consequences and industry manipulation (10.4%), excessive sugar and expertise messages (10.4%), nurturing and expertise messages (9.4%), nurturing and industry manipulation (9.4%), excessive sugar and industry manipulation (9.4%), excessive sugar and humor (9.4%), nurturing and humor (6.6%), industry manipulation and humor (4.7%), health consequences and humor (3.8%), and expertise and industry manipulation (0.9%). There was no overlap observed between expertise messages and humor (0%). Overall, 55.7% of the anti-SSB consumption messages were multi-themed.

Among the pro-SSB messages, the most common theme was feel-good/regular people, followed by feel-good/celebrity and industry. We saw no evidence of an overlap between feel-good/celebrity, feel-good/regular people, and industry (all pairs 0%). That is, none of the pro-SSB appeals were multi-themed.

### 3.4. Style

Table 8 displays the results for message sensation value. A comparison of the mean MSV values revealed that pro-messages were slightly higher than anti-messages. On our 0–1 scale, the difference was (0.38–0.33 =) 0.05. The t value for the comparison of means gave a probability estimate of 0.095.

## 4. Discussion

This study aimed to understand the features of brief, online, English-language videos concerned with SSBs that were produced in the U.S. Though our primary interest was anti-SSB messages, we also sampled pro-SSB advertisements to provide a point of comparison. The analysis of those messages revealed a set of interesting trends, which we turn to next.

### 4.1. Fundamental Features

Our first research question asked about the frequency with which pro- and anti-SSB messages exhibited variation in targets such as consumption, regulation, and industry. The data revealed that almost 9 out of 10 anti-SSB messages took consumption behavior as their persuasive target, whereas only a handful dealt with regulation or policy-related issues. We see several possible reasons for this imbalance. For one, it may reflect a belief among content creators in the value of tackling the SSB problem at the level of individual decision making [33]. The lopsidedness might also result from the fact that citizen-level input into decision making about restrictive policy issues is comparatively rare. In the U.S., for example, only 10 attempts to raise taxes on SSBs through voter referenda were undertaken between 2012 and 2020 [34]. Third, previous research has found a similar unevenness in the news coverage of SSBs in mainstream British papers. Elliot-Green and colleagues report that 81% of the SSB-related stories suggested that SSBs were unhealthy, while only 24% called for policy changes [35]. These findings may be interpreted as evidence of a shared reality among news writers and public health advocates, one in which there is clear agreement on the negative health impact of SSBs, while thinking about policy-level solutions is more diffused. Such a situation calls for a strategic change: public health messages should both educate citizens on the efficacy of policy action and encourage collective efforts toward regulatory change.

A smaller but still substantial number of anti-SSB messages—slightly over one-fifth of our sample—provided a criticism of the beverage industry. This target almost always (90%) co-occurred with consumption. The implicit argument hinged on the undesirability of consuming a product that was marketed in a duplicitous fashion.

It is also informative to ask what was not present in our fundamental features results. The most notable absence may be appeals aimed at persons in the social network of SSB drinkers, appeals that encourage network members to discourage SSB consumption among drinkers. The sole exception was nurturing appeals, which advise parents to limit their children’s consumption. But even these messages were concerned more with controlling access to SSBs than with establishing injunctive norms. To the extent that media campaigns have the potential to produce direct effects via message exposure and indirect effects via interpersonal communication about campaigns [36], the lack of other-oriented appeals may be a consequential omission. While such indirect effects are by no means guaranteed, they would seem to offer an unexplored avenue of influence.

### 4.2. Actor Demographics

The second research question asked how frequently actors of varying gender, age, and ethnicity appear in pro- and anti-SSB messages. We justified the question based on the assumption that this message feature indicated variations in the intended audience. The results showed that both men and women were present in over two-thirds of both anti- and pro-messages. Statistical tests that compared gender proportions across anti- and pro-messages were nonsignificant. Within the set of anti-messages, men and women were present to essentially the same degree (83% vs. 81%). Among the commercial advertisements, however, men were almost universally present (96%), whereas women appeared in only 68% of the messages. This difference echoes Brownbill et al.’s [22] analysis of pro-SSB messaging on Facebook, which found that SSB advertising tilted toward male actors. Both our results and those of Brownbill et al. [22] allow for the conclusion that the beverage industry is engaged in efforts to masculinize consumption, presumably as a means of increasing the bottom line.

The comparison of the proportion of adult, adolescent, and child actors across anti- and pro-messages revealed a significant difference for each of the age categories. Adults appeared in 96% of the industry ads but only 74% of the anti-messages. This pattern reversed for adolescent and child actors, who appeared less frequently in pro- than anti-messages. These differences may, as we suggested, stem from decisions by message creators to target these different audiences. It seems equally plausible, however, to be the product of the different persuasive goals. Anti-SSB messages seek to prevent or extinguish consumption behavior—something that might be achieved by persons of any age deciding to drink less or by parents restricting their children’s intake. In contrast, the beverage industry tries to create or amplify consumption behavior. This aim might be accomplished by selling to children a vision of adult behavior as a means of marketing sugary drinks.

In the U.S., racial and ethnic minority groups are also more likely to consume SSBs than their White counterparts [37] and more likely to suffer the health consequences thereof [38]. These disparities may be the product of the disproportionate exposure by Black and Hispanic youth to SSB advertising on television and in their neighborhoods [39,40]. In our message data, White and Black actors were present in more than 60% of both anti- and pro-messages, whereas Hispanic and Asian actors were seen in 35% or fewer of both message types. If actor ethnicity is a valid indicator of the intended audience, these results are concerning.

To correct known health disparities, we might hope that Black and Hispanic actors would be over-represented in anti-SSB data. But, as noted, Blacks are equally represented vis a vis Whites, not over-represented. The problem is more acute for Hispanics, who are woefully under-represented in anti-SSB messages. And, although there is some evidence that persons of Asian descent consume SSBs in roughly the same proportion as Whites [41], this group is also severely under-represented in our anti-SSB data relative to Whites. If anti-SSB messaging is intended to reduce health disparities resulting from SSB consumption, it is unlikely to succeed if the universe of anti-SSB messages is not better adapted to ethnicity-based audience segments.

### 4.3. Persuasive Themes

All persuasive messages possess some strategy or theme that unites the message elements into a whole. Our third research question was concerned with which themes were present in pro- and anti-SSB messages and how frequently each one appeared. One important finding to emerge from our analysis was that the persuasive themes that appeared in anti- and pro-SSB messages showed no overlap whatsoever. This result resonates with the claim above that anti- and pro-messages differ with respect to the type of behavioral change that they seek: do versus do not. As we show below, the underlying justifications for doing versus not doing differ as well.

Our analysis of anti-messages uncovered six distinct themes, a larger number than has been observed in previous research [23]. This difference may follow from our larger sample of messages. Ordered from most to least, the four most frequently occurring themes were excessive sugar, nurturing, health consequences, and logic.

Given the success of the anti-smoking Truth campaign, which made a heavy utilization of the anti-industry theme [42], it was surprising to find it used so infrequently in anti-SSB campaigns. Still, Donaldson et al. [43] reported that only 38% of their respondents were convinced or very convinced by a message that highlighted the manipulative nature of the advertising strategies used by the soft drink industry. Perhaps yet-to-be-identified context differences between smoking and SSB consumption account for this discrepancy.

Humor occurred least frequently, and this may be for the best: when directly compared to other themes, it is consistently found to be less effective [23,27,44,45]. This does not mean that there is no role for humor in anti-SSB campaigns. It does suggest that humorous PSAs might function best in a supporting rather than leading role [45].

One of the more intriguing findings was that anti-SSB messages typically leverage multiple persuasive themes; over half contained two or more themes. This leads to the concern that multiple themes might overwhelm viewers or muddle the clarity of any one theme. However, research on anti-smoking PSAs found an additive effect for persuasion themes; More themes were associated with lowers levels of smoking prevalence [46]. Still, themes probably cannot be thrown together without regard for compatibility. The most common theme pairing observed in our data was health consequences and nurturing. These strategies were natural partners in messages that touched on the affection and responsibility inherent to the parent–child relationship and the warning of dangers that might befall the child. Themes of logic, industry manipulation, and humor were present in 27% or fewer of the anti-messages. And it is easy to see that logic and humor are incompatible with one another and that, similarly, industry manipulation and humor are mutually exclusive.

All of our pro-SSB messages utilized one and only one theme. Although it is difficult to assess whether this might be a function of our small sample of pro-messages, it does seem to be a sensible result in terms of message content. The co-presence of celebrities and regular people is incompatible. And the feel-good-and-have-fun theme that undergirds SSB consumption is incongruent with a focus on the actions of the beverage industry. Overall, it might be said that there are quite a number of reasons to avoid consuming SSBs but only a few that underwrite increased uptake.

### 4.4. Message Sensation Value

MSV is a stylistic message feature based on objective message components such as visceral images, sound saturation, and special effects [24]. It has been shown to influence persuasion in a variety of ways [25,26]. Accordingly, our final research question asked what the levels of MSV are in anti- and pro-SSB messages and how they compare to other health domains. With respect to the first part of this question, we compared MSV values for pro- and anti-SSB messages. The observed values were 0.38 and 0.32, respectively. They suggested a difference, but at *p* < 0.10, the associated probability value did not meet conventional standards for statistical significance. With regard to effect size, the values of the *d* statistics were smallish by Cohen’s [47] rule of thumb but large by Funder and Ozer’s [48] more recent criteria. If the difference is real, it could be attributable to the financial resources available to the industry. MSV values depend, in part, on the number of cuts and the use of music and sound effects, all of which increase production costs. Such costs are more easily borne by the highly profitable beverage industry.

The second part of RQ4 was concerned with how the MSV values in the current study compared with other health domains. Here, the similarity was striking. Paek et al.’s [27] analysis of 934 anti-smoking video clips that appeared on YouTube produced an MSV score of 0.31 (rescaled to our 0–1 metric). Rhodes et al.’s [28] examination of 487 anti-smoking advertisements showed a value of 0.31 for messages that targeted adolescents and 0.24 for messages that targeted other audience segments (also rescaled). In combination with our findings, these results suggest a fairly narrow band of central tendencies across the message topic and direction of the advocacy. Further research is needed to assess whether or not the naturally occurring variation around these means figures into message effectiveness.

### 4.5. Strengths and Limitations

Our efforts to collect anti-SSB appeals were systematic and rigorous. They were also successful, at least insofar as they produced a sizeable number of messages. It is, however, impossible to assess whether or not these efforts captured the totality of the anti-SSB message universe.

The set of pro-SSB advertisements was the product of two types of systematic sampling. We gathered marketing appeals for six different categories of SSBs. Within these categories, we selected the most popular messages (as determined by search engine rankings). We also chose a set of messages developed by the beverage industry to promote itself. That set of messages might be considered a census given that they comprised the totality of appeals developed by the industry at the time of the research. But, because it was not a probability sample of a population whose parameters are known, we cannot claim that it was representative of the universe of commercial SSB appeals. Nonetheless, the examination of the total set of pro-SSB messages led us to believe that it constituted a meaningful point of reference for the anti-SSB appeals.

## 5. Future Research

The scientific study of any phenomenon begins with description. Indeed, the follow-on goals of science—prediction and control—are achievable only when they stand on a foundation of careful and thorough specification of the thing of interest. While our project is descriptive, it is valuable for the direction it provides to future research. Consider the finding that anti-SSB messages mostly targeted individual-level decision making with respect to consumption to the exclusion of policy-oriented messages. This imbalance means that two persuasive tools that could be working synergistically are not doing so because one is effectively absent. There is evidence that exposure to anti-consumption messages produces positive spillover into support for restrictive SSB policies [48]. Research is needed to test the possibility that the causal arrow can be reversed such that the persuasion that brings about policy change can also indirectly bring about reduced personal consumption.

Relative to White and Black actors, Hispanic and Asian actors were under-represented in anti-SSB messages. One research question that flows easily from this finding concerns the extent to which matching the ethnicity of actors with the ethnicity of message recipients actually produces enhanced persuasion (i.e., decreased consumption). This is widely believed and has been shown to be the case in other health domains [17,18,19,20]. However, empirical research is needed to demonstrate the efficacy of character–audience matching in the context of SSBs. If the predicted effect holds, frontline public health advocates should move toward the creation of anti-consumption messages that more frequently present Hispanic and Asian actors.

Six distinct persuasive themes were identified among the anti-SSB appeals, and they frequently intermingled multiple themes in the same message. The identification of the themes makes possible the obvious next step, that is, evaluating the persuasive efficacy of each. Such research has immediate implications for the allocation of resources in public health agencies responsible for the production of anti-SSB messages. In the process of evaluation, the possible effects of MSV should not be overlooked. Although it has been the focus of research for many years, the analysis of the functional form of the MSV–persuasion association is understudied. And the potential for MSV to interact with one or more of the persuasive themes is untested.

## 6. Conclusions

Our content analysis of anti-SSB messages succeeded in summarizing various features of those appeals. In so doing, it provided a heretofore unseen picture of the collective efforts of U.S. health agencies and advocates to limit SSB consumption via media campaigns. That picture revealed a need for more anti-SSB messaging that is policy-related and industry-focused. Further, anti-SSB appeals should strive for an enhanced representation of minority groups. Finally, future research is needed that explores the persuasive impact of different message properties, most notably variations in persuasive themes and message sensation value. Work along these lines can shed light on important theoretical and applied questions concerning the effectiveness of anti-SSB messages. Such knowledge would constitute a crucial component in a multi-pronged effort to combat SSB consumption.

## Figures and Tables

**Table 1:** Codes and reliability estimates for fundamental message features.

Fundamental Message Features	Codes	*αk*
The Target of the Advocacy		
Consumption	0 = Not consumption 1 = Consumption	0.84
Regulation	0 = Not regulation 1 = Regulation	1.00
Industry	0 = Not industry 1 = Industry	0.77

**Table 2:** Codes and reliability estimates for actor demographics.

Actor Demographics	Codes	*αk*
Gender		
Men	0 = No men present 1 = One or more men present	0.98
Women	0 = No women present 1 = One or more women present	0.88
Age		
Adults	0 = No adults present 1 = One or more adults present	0.87
Adolescents	0 = No adolescents present 1 = One or more adolescents present	0.85
Children	0 = No children present 1 = One or more children present	0.90
Ethnicity		
White	0 = No Whites present 1 = One or more Whites present	0.85
Black	0 = No Blacks present 1 = One or more Blacks present	0.85
Hispanic	0 = No Hispanics present 1 = One or more Hispanics present	0.86
Asian	0 = No Asians present 1 = One or more Asians present	0.81

**Table 3:** Codes and reliability estimates for persuasive themes.

Persuasive Themes	Codes	*αk*
Anti-SSB Messages		
Excessive Sugar	0 = Absent 1 = Excessive Sugar present (e.g., superabundance of sugar)	0.94
Nurturing	0 = Absent 1 = Nurturing present (e.g., parents caring for children)	0.92
Health Consequences	0 = Absent 1 = Health Consequences present (i.e., a description of adverse medical conditions)	0.82
Expertise	0 = Absent 1 = Expertise present (e.g., healthcare professionals)	0.97
Industry Manipulation	0 = Absent 1 = Industry Manipulation present (i.e., claims regarding the duplicity of beverage industry)	0.87
Humor	0 = Absent 1 = Humor present (i.e., attempts to amuse)	0.77
Pro-SSB Messages		
Feel-Good/Regular People	0 = Absent 1 = Lay people present (i.e., non-celebrity actors representing members of the target audience)	0.82
Feel-Good/Celebrity	0 = Absent 1 = Celebrity present (e.g., Anthony Davis)	0.80
Industry Promotion	0 = Absent 1 = Industry Messages present (i.e., illustrations of industry working to provide customer wants)	1.00

**Table 4:** Codes and reliability estimates for message sensation value.

Elements of MSV	Codes	*αk*
Number of Cuts	0 = Low (0–6 cuts) 1 = Moderate (7–14 cuts) 2 = High (15 or more cuts)	0.87
Special Visual Effects	0 = Absent 1 = One or more Special Visual Effects present (i.e., actions beyond the range of human ability)	0.78
Altered Speed of Motion	0 = Absent 1 = Present (i.e., the slowing down or speeding up of an action via technical intervention)	0.84
Intense Images	0 = No Intense Images present 1 = One or more Intense Images present (e.g., damaged body parts, death)	0.92
Sound Saturation	0 = Absent 1 = Present (i.e., discordant or irrelevant audio)	0.93
Music	0 = Absent 1 = Music present	0.91
Sound Effects	0 = Absent 1 = Unusual, non-human sounds (e.g., record scratches, crash noises)	0.90
Visualization	0 = Absent 1 = Visualization present (i.e., imagery used to support or explain concepts presented verbally)	0.91
Surprise Ending	0 = Absent 1 = Surprise ending present (i.e., twist endings or unexpected second half)	0.68
Total MSV		0.86

**Table 5:** Fundamental message features.

	Direction of Advocacy	
Target of Advocacy	Anti-SSB (*k* = 112)	Pro-SSB (*k* = 29)
Consumption	94.6% (106)	100% (29)
Regulation	3.6% (4)	0% (0)
Industry	17.9% (20)	24.1% (7)

*Note.* Proportions are computed column-wise. Because the codes are non-exclusive within the direction of the advocacy, within-column frequencies exceed the number of messages (*k*) in our dataset (i.e., *k* = 112 anti-SSB messages, and *k* = 29 pro-SSB messages).

**Table 6:** Actor demographics by direction and target of advocacy.

	Direction and Target of Advocacy		
Demographic Feature	Anti-SSB Consumption (*k* = 86)	Pro-SSB Consumption and Industry (*k* = 25)	Anti Minus Pro
Gender			
Men	84.8% (73)	96.0% (24)	−11.1%
Women	81.4% (70)	68.0% (17)	13.4%
Age			
Adults	69.8% (60)	96.0% (24)	26.2% **
Adolescents	57.0% (49)	28.0% (7)	−29.0% *
Children	66.3% (57)	32.0% (8)	34.3% **
Ethnicity			
White	62.8% (54)	84.0% (21)	−21.2% *
Black	68.6% (59)	68.0% (17)	0.6%
Hispanic	39.5% (34)	32.0% (8)	7.5%
Asian	27.9% (24)	16.0% (4)	11.9%

*Note.* Proportions are based on the total number of messages (*k*) in each column. Messages without actors or with non-human actors were removed from both columns. Probabilities are derived from z-tests of proportions. * *p* < 0.05. ** *p* < 0.01.

**Table 7:** Persuasive themes by direction and target of advocacy.

	Direction and Target of Advocacy		
Theme	Anti-SSB Consumption (*k* = 106)	Pro-SSB Consumption and Industry (*k* = 29)	Anti Minus Pro
Excessive Sugar	44.3% (47)	0% (0)	44.3% ***
Nurturing	43.4% (46)	0% (0)	43.4% ***
Health Consequences	43.4% (46)	0% (0)	43.4% ***
Expertise	25.5% (27)	0% (0)	25.5% **
Industry Manipulation	21.7% (23)	0% (0)	21.7% **
Humor	14.2% (15)	0% (0)	14.2% *
Feel-Good/Regular People	0% (0)	44.8% (13)	−44.8% ***
Feel-Good/Celebrity	0% (0)	31.0% (9)	−31.0% ***
Industry Promotion	0% (0)	24.1% (7)	−24.1% ***

*Note.* Proportions within columns are based on the total number of messages (*k*) in that column. Probabilities are derived from z-tests of proportions. * *p* < 0.05. ** *p* < 0.01. *** *p* < 0.001.

**Table 8:** Message sensation value by direction and target of advocacy.

Direction and Target of Advocacy				
Anti-SSB Consumption (*k* = 106)		Pro-SSB Consumption and Industry (*k* = 29)		
*M*	*SD*	*M*	*SD*	*d*
0.33	0.14	0.38	0.15	0.34

*Note*. *t* (133) = 1.68, *p* < 0.10.

## Data Availability

The data presented in this study are available on request from the corresponding author.
